# Harnessing the self-harvesting capability of benthic cyanobacteria for use in benthic photobioreactors

**DOI:** 10.1186/2191-0855-1-19

**Published:** 2011-07-18

**Authors:** Diane Esson, Susanna A Wood, Michael A Packer

**Affiliations:** 1 Cawthron Institute, Private Bag 2, Nelson, 7001, New Zealand; 2 Cambridge Institute for Medical Research, University of Cambridge, Wellcome Trust/MRC Building, Hills Road, Cambridge, CB2 0XY, UK

## Abstract

Benthic species of algae and cyanobacteria (i.e., those that grow on surfaces), may provide potential advantages over planktonic species for some commercial-scale biotechnological applications. A multitude of different designs of photobioreactor (PBR) are available for growing planktonic species but to date there has been little research on PBR for benthic algae or cyanobacteria. One notable advantage of some benthic cyanobacterial species is that during their growth cycle they become positively buoyant, detach from the growth surface and form floating mats. This 'self-harvesting' capability could be advantageous in commercial PBRs as it would greatly reduce dewatering costs. In this study we compared the growth rates and efficiency of 'self-harvesting' among three species of benthic cyanobacteria; *Phormidium autumnale*; *Phormidium murrayi *and *Planktothrix sp*.. *Phormidium autumnale *produced the greatest biomass and formed cohesive mats once detached. Using this strain and an optimised MLA media, a variety of geometries of benthic PBRs (bPBRs) were trialed. The geometry and composition of growth surface had a marked effect on cyanobacterial growth. The highest biomass was achieved in a bPBR comprising of a vertical polyethylene bag with loops of silicone tubing to provide additional growth surfaces. The productivity achieved in this bPBR was a similar order of magnitude as planktonic species, with the additional advantage that towards the end of the exponential phase the bulk of the biomass detached forming a dense mat at the surface of the medium.

## Introduction

Algal productivity is up to an order of magnitude greater than that of most terrestrial crops making them a promising feedstock for many industrial processes (Packer 2009; Stephens et al. 2010). Atmospheric carbon dioxide is fixed into biomass by photosynthetic activity as part of the biogeochemical cycling of carbon. Biofuel production offers potential recycling of anthropogenically-released carbon from fossil stores. Their high productivity has therefore led to a great deal of interest in commercial-scale algal production. Despite the promise, and a good deal of hype, several hurdles remain before economic commodity-scale algal farming becomes a reality. Two significant restraints are a lack of cost-effective bioreactor technology and efficient harvesting (dewatering) techniques (Norsker et al. 2011). There are two main ways of growing algae; open ponds and enclosed bioreactors systems (Bosma et al. 2007; Heubeck and Craggs 2007; Packer 2009) both of which are generally used for planktonic species where the cells are in suspension. Very little attention has been given to developing growth systems for benthic species, those that require a surface to grow on (Mulbry et al. 2006).

Cyanobacteria are an ancient group of prokaryotic organisms that have evolved many unique physiological adaptations including; the ability to scavenge limiting resources (Kim et al. 2010; Carlsson et al. 2007), the presence of distinct and extremely efficient light harvesting complexes known as phycobilisomes (Grossman et al. 1993), the ability to store significant amounts of nitrogen and phosphorous in excess of their immediate requirements and a tolerance to a wide range of physiochemical conditions (Whitton and Potts 2000). These features make them particularly amenable for commercial growth, and they are presently one of the few species of algae that are successfully grown commercially (Carlsson et al. 2007). Many species of cyanobacteria grow attached to a surface in aquatic environments, at times forming expansive and thick benthic mats (Heath et al. 2010). As these mats develop oxygen bubbles become entrapped amongst filaments and associated mucilage. If undisturbed at a certain point in their growth cycle the mats become positively buoyant, detaching from the growth surface and forming thick floating mats. This feature has been observed in the environment and in our cultures (Wood et al. 2010). This 'self-harvesting' capability would be advantageous in commercial applications as it has the potential to greatly reduce the dewatering cost of biomass harvesting.

In this manuscript we describe experiments aimed at providing data for the development of a novel benthic PBR (bPBR) system. Comparative growth experiments were undertaken on three species of benthic cyanobacteria and their productivity compared with those of planktonic algae and cyanobacteria. The species with the fastest growth and most efficient 'self harvesting' capability was selected for further investigation. A commonly used cyanobacterial growth media (MLA) was optimized to maximize biomass production. We then investigated alternative bPBR systems with the ideal that productivity would be similar to planktonic species but that the benthic cyanobacteria would 'self-harvest'. The released mats would then potentially require much less energy to collect than current techniques used to harvest planktonic species.

## Materials and methods

### Cultures

Three strains of benthic cyanobacteria were used in this study; all are now present in and are publically from the Cawthron Institute Culture Collection of Microalgae^a^. *Phormidium autumnale*, CAWBG26, was isolated from the Hutt River, Wellington, New Zealand and is a common species found throughout New Zealand rivers. During stable flow conditions, *Ph. autumnale *mats proliferate, at times forming expansive black/brown leathery mats across large expanses of river bed (Heath et al. 2010). *Phormidium murrayi*, CAWBG22, was isolated from a small tarn in the Red Hills, Nelson, New Zealand, where it formed extensive mats. Prior to this identification, *Ph. murrayi *was known only from Antarctica (Heath *et al*. 2010). The final strain, CAWBG59, was isolated from a benthic mat in the Waitaki River, Christchurch, New Zealand, and has tentatively been identified as a *Planktothrix *sp. (Wood et al. 2010).

### Growth experiments

Aliquots (4.2-4.8 mg wet mass) of each strain were added to 50 mL clear polystyrene bottles (pottles) (Biolab, New Zealand) containing 30 mL of MLA medium (Bolch and Blackburn 1996). For each strain 15 pottles were set up, enabling collection in triplicate at five time points. The samples were grown at 18°C ± 1°C under 36.8 μE.m^2^.s^-1 ^of light from cool white fluorescent tubes arranged above the cultures on a 12:12 h light:dark regime. Light levels were assessed by averaging ten measurements (LI_185B, LiCor Inc., USA). Cultures were static. After five days, triplicate samples were harvested. Media was aspirated off and pottles were dried at 50°C for 26.5 h. Each pottle was weighed, thoroughly cleaned and dried and re-weighed to calculate the increase in dry mass. Samples were harvested approximately every four days. The growth was noticeably slower in CAWBG22 and harvesting was extended to eight days.

### Media optimisation experiment

*Phormidium autumnale *(CAWBG26) was selected for an experiment to optimize MLA media under conditions described above. MLA is comprised of NaNO_3 _(2.00 mM), NaHCO_3 _(2.019 mM), MgSO_4 _7H_2_O (200.43 μM), CaCl_2 _2H_2_O (200 μM), K_2_HPO_4 _(199.77 μM), NaEDTA (11.7 μM), H_2_SeO_3 _(10.00 μM), H_3_BO_3 _(39.95 μM), MnCl_2 _4H_2_O (18.19 μM), FeCl_3 _6H_2_O (5.85 μM), CuSO_4 _5H_2_O (40.1 pM), ZnSO_4 _7H_2_O (76.5 pM), CoCl_2 _6H_2_O (79.86 pM), Na_2_MoO_4 _2H_2_O (24.8 pM), Biotin (0.05 μg/L), Vitamin B_12 _(0.05 μg/L) and Thiamine HCl (100 μg/L) (Bolch and Blackburn 1996). The concentrations of the following elements were modified: nitrogen, iron, calcium, and selenium. Nitrogen amounts were varied due to the element's role as a major macronutrient, the abundance of which is often responsible for algal blooms (Oliver and Ganf 2000). Iron was chosen as it plays an important role in cellular functions, especially redox reactions, and carbon and nitrogen reduction (Rueter and Petersen 1987). Iron has also been shown to stimulate growth in many species (Paerl et al. 1994; Hyenstrand et al. 2001; Li et al. 2009; Swingley et al. 2005). Calcium was selected because in most biological systems it's heavily involved in cellular signaling and following this may be important in regulating responses to environmental variables (Norris et al. 1996; Smith 1995; Giraldez-Ruiz et al. 1997). The selenium concentration was altered in the attempt to replicate the natural environment of the cyanobacteria in New Zealand as soils in this country are selenium deficient.

Nitrogen in standard MLA medium is at 2.00 mM concentration (representing a 10:1:1 ratio of nitrogen: phosphorus: potassium). In the media manipulation experiments nitrogen was increased to 4.00 mM, providing an experimental 20:1:1 NPK ratio. Iron was increased from 5.85 μM to 11.7 μM (2× original concentration) and 58.5 μM (10× original concentration). Calcium was increased from 199.98 μM to 400 μM (2× original concentration) and 4.0 mM (20× original concentration). Selenium, in standard MLA media at a concentration of 10 μM, was experimentally removed and also increased to 20 μM (2× original concentration) and 100 μM (10× original concentration). Each experiment was set up in triplicate and harvested at five time points, approximately four days apart, as described above.

### Geometry and surface area optimisation experiment

**Figure 1:**
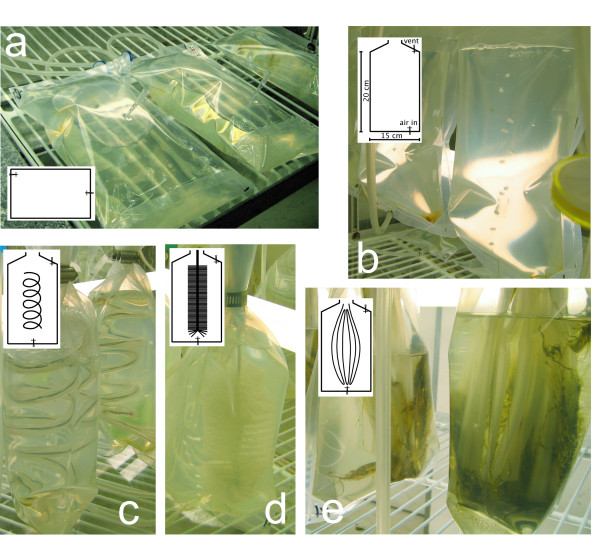
**Benthic PBR geometries compared**. *Phormidium autumnale *(CAWBG26) was grown in the bags as described in the Materials and Methods section. panel a, horizontal PBR bags; panel b, vertical PBR bags; panel c, silicone tubing added in a coiled spiral (using a flexible wire inserted within to shape); panel d, a PVC bottle brush inserted into the PBR; panel e, 'looped silicone' where silicone tubing was folded back upon itself several times and pinched together at the bottom using a silicone tie. These loops were able to move about in the air bubble stream. Dimensions shown on the insert for panel b are the same for the other geometries.

*Phormidium autumnale *(CAWBG26) was used in further experiments to explore bPBR geometry and configurations that might be suitable for large-scale growth. Aliquots (39.7-46.6 mg wet mass) of CAWBG26 were used to inoculate plastic bags with the dimensions of 15 cm × 24 cm made of heat-sealed 250 μm thick crystalline polyethylene (Aperio Plastic Ltd, Christchurch, New Zealand) with approximately 600 cm^2 ^of usable surface area. The bPBR bags were filled with 1 L optimized media (10 × iron MLA, as described above) and bubbled through with air, with no additional carbon dioxide (CO_2_), from the base at a rate of 200 mL min^-1^. Light and temperature were as for previous experiments. Previously when growing benthic species the above bags had been positioned horizontally in a 'lay flat' position (Figure 1a). We compared this configuration with the same bags hanging vertically (vertical, Figure 1b) and also to vertical bags that had additional growth surface areas by adding coiled silicone tubing (coiled spiral, Figure 1c), a PVC bottle brush (Silva-Aciares and Riquelme 2008), Figure 1d), or loops of silicone tubing (Figure 1e). The coiled spiral silicone supplied ~220 cm^2 ^extra surface area, the bottle brush an additional ~250 cm^2 ^surface area, and the silicone loops ~264 cm^2 ^extra surface area. Each experiment was set up in triplicate and harvested 36 days after inoculation. Date of detachment was recorded.

Statistical analysis was performed with GraphPad Prism 5.0 software for Mac OS-X.

## Results

Five days after inoculation, both *Ph. autumnale *(CAWBG26) and *Planktothrix *sp. (CAWBG59) had filament growth extending from the initial inoculation site across the walls of the culture bottles. By the thirteenth day of growth, all available growth surfaces were covered and there was a visible ring of filaments at the surface of the media. In addition, CAWBG26 was spreading across the media surface. CAWBG59 and CAWBG26 continued to grow across the media surface and gradually peeled from the pottle sides until complete detachment. In contrast, *Ph. murrayi *(CAWBG22) increased in bulk at the initial inoculation site, with very little spread of filaments across the bottom of the culture bottle.

**Figure 2:**
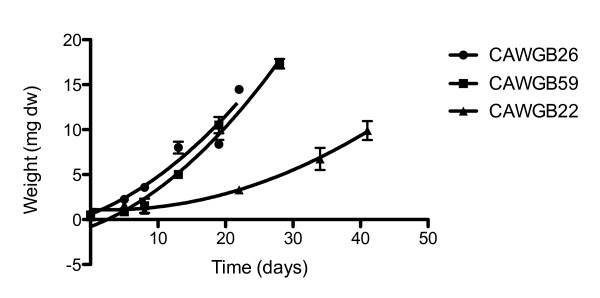
**Comparison of growth of three different benthic cyanobacteria species (*Phormidium murrayi *(CAWBG22), *Ph. autumnale *(CAWBG26), *Planktothrix *sp. (CAWBG59)) in MLA media**. Values represent mean ± standard error (*n = 3*) and the lines of best fit are shown.

CAWBG26 detached on day 22 with an average final biomass of 14.5 mg, CAWBG59 detached on day 28 with an average final biomass of 17.3 mg. CAWBG22 had not detached by the end of the experiment (day 41) and had the smallest average biomass of 9.9 mg (Figure 2). While harvesting did not require the removal of the cyanobacterial mats (the water was aspirated out of the culture bottle), the texture of the different species' mats was noted. Specifically, CAWBG26 formed a cohesive mat which was easily picked up with tweezers, whereas the filaments of CAWBG59 were not tightly held together and harvesting was difficult. CAWBG22 never morphed into a single mat.

**Figure 3:**
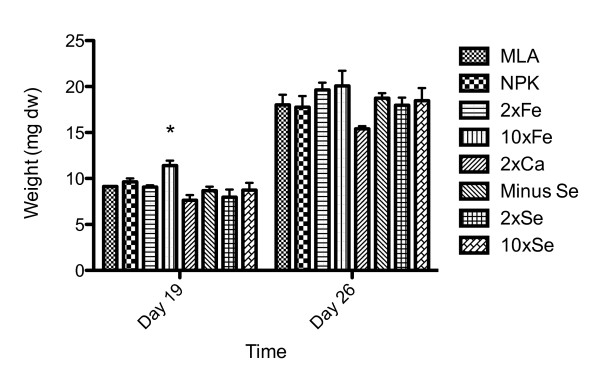
**Media optimization of *Phormidium autumnale *(CAWBG26)**. Values represent mean ± standard error (*n = 3*), * indicates statistical significance (*P *= 0.0148, Student's t-test). NPK, 20:1:1 nitrogen: phosphorus: potassium ratio; Fe, iron; Ca, calcium; Se, selenium. Earlier time points were also measured (day 5, 8 and 13, data not shown) where there was no significant difference.

CAWBG26 was used for all further experiments aimed at optimizing growth in MLA media and testing bPBR geometry. Changing the concentrations of selected MLA ingredients had a statistically significant affect on the mean weights of the CAWBG26 biomass on day 19 (Figure 3, one-way analysis of variance, *P *= 0.0055). Differences in biomass were not significant on day 26, with the exception of the media containing 10 × iron (day 19) when compared to data for the control medium (MLA) with a *P *value of 0.0148 (Student's t-test). A further advantage of the 10 × iron media was that CAWBG26 was observed to fully detach at least three days ahead of the other conditions. Interestingly, when grown in media with 2 × and 20 × calcium, CAWBG26 had not fully detached by day 26.

**Figure 4:**
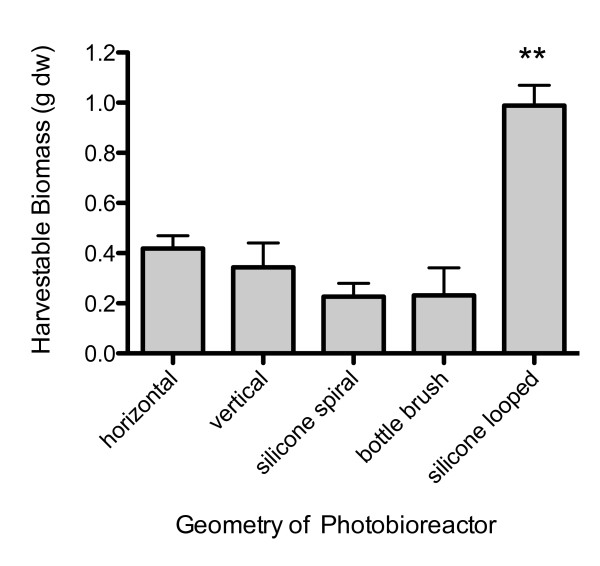
**Harvestable biomass of *Phormidium autumnale *(CAWBG26) after 36 days of growth during geometry and surface area optimization experiments**. Values are mean ± standard error (*n = 3*), ** indicates highly statistical significance (P = 0.0003).

The 10 × iron medium was used in upscaled experiments with varying substrates, designed to explore optimal bPBR geometry. In preliminary experiments we trialled a range of different supports (PVC, vinyl, polystyrene, polyester and silicone) with horizontal bags using CAWBG26 (data not shown). These data indicated that CAWBG26 adhered to and then detached best from PVC and silicone, and these supports were selected for the experiments to test different formats for pPBR geometry. Total biomass was significantly greater in the bPBR bags containing loops of silicone (0.99 g per bPBR) than either bPBRs not containing additional growth surfaces (0.34 g and 0.42 g for the vertical and horizontal systems respectively), silicone coiled spirals or PVC bottle brush bPBRs (average of 0.23 g for both) (Figure 4). The differences in biomass produced between all other bPBR geometries were not statistically significant from each other as a group (one way ANOVA, *P *= 0.3436), nor was any one of these other geometries statistically significantly different from any other by Student's t-test.

**Figure 5:**
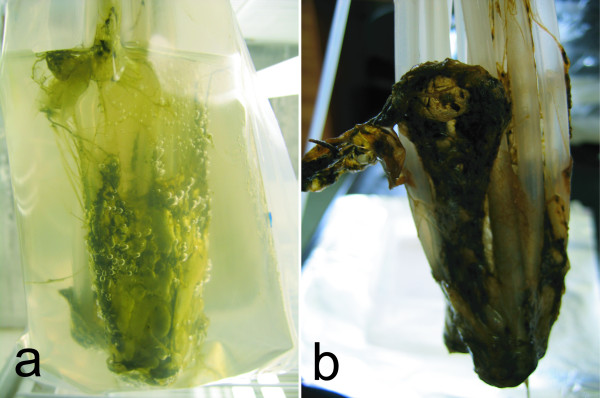
**Photograph illustrating of *Ph. autumnale *biomass extraction at day 36**. Panel a, fully grown culture at the end of the experiment showing coverage of the silicone loops inside the PBR; panel b, the loops removed showing the ease of the cyanobacterial biomass slipping off as one mass. If the cultures were left untouched a large mat would "self-release" from the silicone loops rising to the surface of the PBR leaving cyanobacterial filaments behind which were sufficient to re-innoculate the next culture.

The majority of the biomass in the looped silicone bPBR was attached to the base of the tubing and it was very fibrous in composition. This allowed most of it to be easily harvested (Figure 5). Following harvesting a small amount of the cyanobacteria remained attached at the base, which was the first region in the bPBR to be colonised. The amount remaining may serve as an innoculum for a next cycle of growth. Biomass was also easily extracted from the empty horizontal and vertical PBR bags as it was observed that these species only loosely associated with the polyethylene bag itself. In these PBRs without additional growth surfaces the tip of the silicone tubing used to deliver air bubbles was normally the focus of the initial colonization. Only a small proportion of cyanobacterial filaments were removable from the bristles of the PVC bottlebrush.

## Discussion

Growth rates in cultures of planktonic species can be monitored via regular sampling and cell counts. These methods cannot be applied to benthic mat-forming species without disturbing their mat-like growth. In this study we developed a method which involved inoculating multiple 30 mL cultures with approximately 7 mg of benthic cyanobacterial strains and harvesting replicate containers regularly through a growth curve. Leflaive and colleagues (Leflaive and Ten-Hage 2009) used a similar approach with the filamentous cyanobacterium *Uronema confervicolum *to study allelopathic interactions in complex benthic communities. Our approach requires careful experimental design with many replicate cultures to have sufficient data points over important phases of a growth curve. Despite this difficulty it was found to work well enabling accurate biomass comparison for the work presented here.

Of the three species tested, *Ph. autumnale *(CAWBG26) was found to have the fastest growth rate, highest final biomass and its detachment from surfaces made for easy harvesting. In contrast, the 'slimy' consistency of the *Planktothrix *sp. (CAWBG59), made this species difficult to handle, whereas *Ph. autumnale *(CAWBG26) form cohesive dense mats, ideal for simple harvesting. This feature in combination with CAWBG26's faster growth during the exponential phase make this species amenable as a promising candidate for the development of large-scale bPBR systems. In the media optimization assay, the final *Ph. autumnale *biomass did not differ significantly between media although at day 19 the biomass in the 10 × iron media was significantly greater than all other media combinations trialed. This result is compatible with previous studies showing cyanobacterial growth to be enhanced by the presence of iron (Paerl et al. 1994; Hyenstrand et al. 2001; Li et al. 2009; Swingley et al. 2005). Additionally, the self-detachment of *Ph. autumnale *was earliest in the 10 × iron media.

The frontal area of filled 1 L bPBRs used in this work is approximately 0.02 m^2^. For the horizontal configuration, the standing biomass at the end of the experiment was 21.0 g.m^-2 ^which was increased considerably in the looped silicone bPBR configuration to 49.5 g.m^-2^. This addition more than doubled the biomass with only a 1.44 fold increase in available surface area. The increase in biomass was not significant in the other bPBR geometries with similar increases in available surface area, suggesting that geometry and/or nature of material of the surface area is important. If this laboratory-determined productivity in this bPBR over the 36 day experiment, could be scaled without light loss and the effects of variable environmental conditions, it would equate to ~5 t.Ha^-1^.yr^-1^. Although of a similar order of magnitude, this is about seven-fold lower than for mixed planktonic species in open raceway pond systems without CO_2 _injection (Heubeck and Craggs 2007) and considerably lower than projections of enclosed bPBRs for planktonic species on similar scales (Bosma et al. 2007; Packer 2009).

Future experiments could address some factors potentially affecting productivity, in the batch experiments presented here, media flow was not continuous (which generally results in higher productivity), additional CO_2 _over the amount present in air was not added and the light level of 36.8 μE.m^2^.s^-1 ^is low compared with average outdoor light. The self-detachment after maturity of this species in this bPBR format facilitating easy harvesting may make up for a lower productivity in downstream dewatering costs. Furthermore, it was observed that after initial detachment from the growth surface, small quantities of *Ph. autumnale *filaments remained in the bPBR and these resumed the growth cycle, suggesting a potential for continuous growth upon nutrient renewal. The efficacy of this would have to be trialed in larger-scale outdoor pilot experiments. Other benefits of the benthic PBR geometry may counterbalance the lower productivity seen here versus planktonic systems in large-scale operations if straight biomass is not the goal, for instance in integrated waste removal, co-electricity (or hydrogen of hydrogen peroxide) or higher value product operations.

Most benthic cyanobacteria are integral members of biofilms, which are complex communities of autotrophic and heterotrophic, eukaryotic and prokaryotic organisms. Future work could also compare the productivity of a single species, as was undertaken in this work, with that of multispecies biofilms, where the stability of the community and allelopathic and positive interactions between the species could affect overall productivity.

Materials suitable as growth surfaces could be further investigated. Here several plastics were examined and it was found that silicone tubing was an effective surface that allowed easy harvesting, but it is likely to be too expensive to be used on a very large scale for commodity products. Materials that allow light to penetrate even if translucent, as opposed to transparent could actually help to deliver light to the algae attached to it, potentially representing a partial engineering solution to the so-called 'light saturation effect' problem (Hallenbeck and Benemann 2002). Benthic cyanobacteria may also offer advantages for use in microbial fuel cells (MFCs), bioelectrochemical systems (BES) that exploit biological catalytic processes for the either the generation of electrical power or accumulation of useful chemicals (such as hydrogen gas or hydrogen peroxide) by releasing electrons from organic substrates by oxidation (Rosenbaum et al. 2010). To date most work on pMFCs has been carried out with such planktonic species like *Synechocystis *sp. and *Anabaena *sp. We hypothesize that benthic species would carry out direct electron transfer (DET) more efficiently than planktonic species in pMFCs because of their more intimate interaction with the surface they grow on. If this is true then using surfaces in the bPBR that are also electrically conductive would be necessary.

The experiments here show that bPBR geometry has a major influence on the productivity of benthic cyanobacteria and that this productivity can be at similar levels to established planktonic algal production systems. Benthic cyanobacteria have many qualities that are suitable for large-scale commerical use and this knowledge makes available a new and diverse group of organisms for algal biotechnology applications. The self-harvesting and self-inoculating aspects of benthic cyanobacteria observed in this work may avail well to a continuous system helping to overcome the major issue of dewatering in commodity production from microalgae. Marine benthic species should be investigated as well as a wider range of materials and configurations to act as growth surfaces. For instance in this work the individual bristles of the PVC bottle brush insert were much finer than the ~7 mm silicone tubing used for other bPBR inserts. This may have influenced the the harvestability of these cultures. In addition to enclosed bPBR technology that this work focused on, providing extra growth surfaces for benthic algae in open systems such as raceway ponds could be explored which might more closely mimic the lotic systems that these benthic species naturally occur in.

## Competing interests

The authors declare that they have no competing interests.

## Endnotes

^a ^http://www.cawthron.org.nz/aquatic-biotechnologies/micro-algae-culture-collection.html
